# Report of Cases

**Published:** 1869-10

**Authors:** Theo. N. Lescher

**Affiliations:** Registrar


### Report of Cases.

Under treatment in the Elmira Eye & Ear Institute, for the Quarter ending October 1st, 1869:

| | |
|---|---|
| Under treatment for Granular Lid | 10 |
| " " " Scrofulous Ophthalmia, | 4 |
| " " " Gonorrhœal " | 6 |
| " " " Purulent " of Infants | 4 |
| " " " Syphilitic Iritis, | 4 |
| " " " Pannus, | 2 |
| " " " Rheumatic Ophthalmia, | 2 |
| " " " Glaucoma, | 2 |
| " " " Catarrhal Ophthalmia | 8 |
| " " " Simple Conjunctivitis, | 5 |
| " " " Suppurative Keratitis, | 4 |
| " " " Meibomitis | 4 |
| " " " Astigmatism | 3 |
| " " " Opacity of Cornea, | 4 |
| " " " Incised wound of Cornea, | 6 |
| " " " Otorrhœa, | 2 |
| " " " Deafness, | 2 |
| " " " Catarrh and Disease of Throat | 8 |
| " " " Fracture of Fore Arm, | 2 |
| " " " Appoplexy, | 1 |
| " " " Spermatorrhœa, | 10 |
| Operated upon for Cataract, | 6 |
| " " " Iridectomy, | 6 |
| " " " Corneal Staphyloma | 2 |
| " " " Entropium, | 3 |
| " " " Extirpation of Eye, | 2 |
| " " " Pterygium, | 2 |
| " " " Strabismus, (Cross Eye,) | 4 |
| " " " Obstruction Lachrymal Duct, | 3 |
| " " " Club Foot | 1 |
| " " " Cleft Palate | 2 |
| " " " Removal of Tumors, | 8 |
| " " " Hœmorrhoids | 2 |
| " " " Stone in Bladder, | 1 |
| " " " Amputation Scirrhus Breast, | 1 |
| " " " Hernia | 1 |
| " " " Nasal Polypii, | 4 |
| " " " Epithelioma, lower lip, | 1 |
| Insertion of Artificial Eyes, | 6 |
| Minor Surgical Operations, | 9 |
| Total number of Cases, | 158 |

THEO. N. LESCHER, Registrar.

